# Evaluation of activities of daily living following pseudophakic presbyopic correction

**DOI:** 10.1186/s40662-016-0067-1

**Published:** 2017-01-19

**Authors:** Georgios Labiris, Panagiota Ntonti, Maria Patsiamanidi, Haris Sideroudi, Kimon Georgantzoglou, Vassilios P. Kozobolis

**Affiliations:** 1 Department of Ophthalmology, University Hospital of Alexandroupolis, 68100 Dragana, Alexandroupolis, Greece; 2 Eye Institute of Thrace, Alexandroupolis, Greece

**Keywords:** Cataract, Presbyopia, Monovision, Multifocal intraocular lenses, Activities of daily living

## Abstract

**Background:**

Pseudophakic presbyopic correction is among the prevalent methods for regaining near vision capacity. The purpose of this study is to compare the impact of pseudophakic monovision correction and multifocal lens implantation on the performance in a series of activities of daily living (ADL) of presbyopic patients.

**Methods:**

An ADL research framework (10 ADLs) was constructed and validated in a sample of patients divided into three validation groups according to their near visual acuity. Sixty-two participants that underwent mini-monovision (MoG) cataract extraction and 60 that underwent bilateral multifocal lenses implantation (MfG) populated study groups and addressed the ADLs. Binocular uncorrected distant (BdUVA) and near (BnUVA) visual acuity were associated with ADL scores and with subjective satisfaction using the VF-14 questionnaire.

**Results:**

Test-retest reliability [all Intraclass Correlations Coefficients (ICC) >0.90] and construct validity (all *p* < 0.05) tests indicated sufficient psychometric performance of the ADL framework. Both study groups presented comparable mean ADL scores (*p* = 0.07) however, MoG patients had lower performance in demanding ADLs (*p* = 0.02). ADL scores demonstrated significant correlation with BnUVA (r^2^ = −0.67, *p* < 0.01) VF-14 scores (r^2^ = 0.53, *p* < 0.01).

**Conclusions:**

Both methods provide sufficient near vision capacity for the majority of activities of daily living. However, only multifocal lens implantation can address demanding near vision tasks.

**Trial registration:**

ClinicalTrials.gov Identifier: NCT02431156.

## Background

Presbyopia is an age-related visual disorder that results in a gradual inability to perform activities that require near vision. It is common for emmetropic populations above 40 years old; eventually almost everyone will demonstrate a variable amount of near-vision impairment [1]. Projection studies suggest that by 2050 about 1.8 billion people will have presbyopia [2]. These people are most likely to experience limited career options, reduced productivity and self-esteem [3–5]. Unfortunately, as with untreated cases, conventional presbyopic spectacles are also associated with negative impact on the quality of life (QoL) [6, 7].

Pseudophakic monovision corrections attempt to address presbyopia in those patients who either have already developed cataract or their associated refractive errors and/or ocular biomechanics exclude laser-assisted corrections, intracorneal implants or multifocal lenses implantation. Recently, our group published the outcomes of a new pseudophakic mini-monovision technique with variable bilateral myopic defocus (MM-VD) reporting high patient satisfaction levels and spectacle independence [8]. In accordance to former similar studies, subjective postoperative functional capacity was measured by the popular VF14 questionnaire [9].

Traditionally, QoL instruments (among them, the NEI-VFQ25, NEI-RQL42 and VF14) have been developed for clinical and research settings as a convenient, self-reported assessment of visual capacity [9–11]. However, despite the popularity of QoL instruments, certain concerns have been raised regarding their psychometric performance as a valid index of visual capacity. Therefore, the prevalent instruments are constantly updated in order to improve their reliability and validity [12].

Ideally, any functional incapacity should be objectively evaluated for each patient in his/her home or working environment, by careful assessment of his/her performance in specific activities of daily living (ADL). However, the inconsistent methods of such data collection make them unsuitable for comparative studies. To provide a common data collection methodology, simulated environments have been developed for a series of chronic diseases where study participants are objectively evaluated in specific ADLs [13, 14]. Unfortunately, a thorough systematic review of the international literature returned no published reports on ADL performance following presbyopic corrections.

Within this context, the primary objective of this study was to evaluate the objective performance in a series of ADLs of patients who had undergone pseudophakic presbyopic surgery either with MM-VD or bilateral multifocal lenses implantation. Among the objectives of the study were: a) to develop a valid framework of ADLs for presbyopic patients, and, b) to compare ADL scores with subjective reports from the VF-14 instrument.

## Methods

### Participants

Participants were recruited from the Cataract Service in a consecutive-if-eligible basis, and randomly populated two study groups: a) MoG study group (participants that underwent uncomplicated lens-extraction surgery with MM-VD), and, b) MfG study group (participants that underwent uncomplicated lens-extraction surgery with bilateral multifocal intraocular lens implantation). The exclusion criteria for both groups included preoperative manifest astigmatism above one diopter (D), glaucoma, IOP-lowering medications, former incisional surgery, former diagnosis of corneal or fundus disease, diabetes, autoimmune, mental diseases, or the inability to perform ADL tasks for reasons not related to visual capacity.

### Surgical technique

All operations were performed by the same surgeon (G.L.) in a consistent manner using the Alcon Infiniti VisionSystem platform (80% continuous amplitude with 350 mmHg vacuum limit and 40 ml/min aspiration flow rate), as described previously [15]., Pupils were dilated with Tropicamide 0.5% (Tropixal, Demo, Greece) and Phenylephrine Hydrochloride 5% (Phenylephrine, Cooper, Greece). The periorbital skin and lids were cleaned and the conjunctival cul-de-sac was irrigated with povidone iodine (Betadine). Patients received topical anesthesia with propacaine hydrochloride 0.5% drops (3 drops prior to surgery). A 2.2 mm, superior-temporal or superior-nasal (eleven o’clock), self-sealing, clear-cornea incision was done and sodium hyaluronate 3.0%, chondroitin sulfate 4.0% (dispersive OVD), and sodium hyaluronate 1.0% (cohesive OVD) (Duovisc) were used at different phases of the operation [16]. Capsulorrhexis was performed with forceps and hydrodissection with BSS. MoG participants received the foldable aspheric acrylic intraocular lens Acrysof SN60WF (Alcon) in the capsular bag, targeting −0.50 D in the dominant eye and −1.25 D in the non-dominant eye. Ocular dominance was assessed using the hole-in-the-card test. MfG participants received the Acrysof IQ Restor (Alcon) with add +2.50 D for near vision in the bag, as well. For all MfG subjects, refractive target was set at +0.25 D for both eyes.

### Construction of activities of daily living. Exploratory study

Among the priorities of the study was to identify those ADLs that require near vision and better reflect visual capacity. The literature review regarding a validated methodology on ADL performance following presbyopia correction was not found. Thus, an exploratory interview study was carried out to ascertain a baseline for the ADL development. A panel consisting of three refractive surgeons and a psychologist were recruited for the exploratory study. A number of ADLs that require near vision were constructed. A sample of patients were interviewed in which they performed the ADLs. The interviews were analyzed and the findings served as the basis for identifying those ADLs that would be operationalized and be used in a universal validated methodology for assessing presbyopia. The final list consisted of ten ADL tasks: Phone Book Search (PBS), Supermarket receipt (SR), Book reading (BR), Cellular message (CM), Cellular entry search (CES), Reading computer screen (RCS), Drops bottle reading (DR), Subtitles reading (SuR), Open door test (ODT), and, Screwdriver test (ST). A detailed description of ADL tasks is presented in Table 1.

**Table 1:** Detailed description of Activities of Daily Living (ADL)

ADL	Description
Phone Book Search (PBS)	Patient is required to find and read a specific entry in a regular phonebook catalog within 60 s with task lighting^a^. PBS score = Task duration. Perceived difficulty is evaluated.
Supermarket receipt (SR)	Patient is required to read a typical supermarket receipt (monospaced Sanserif font) within 20 s with task lighting^a^. ADL score is derived using the formula SR = task duration + (number of errors × 2). Perceived difficulty is also evaluated.
Book reading (BR)	Patient is required to read a chapter in a novel with task lighting^a^. ADL score is derived using the formula BR = task duration + (number of errors × 2). Perceived difficulty is also evaluated.
Cellular message (CM)	Patient is required to read an SMS on a 4-in. cellular phone^b^ in an environment with ambient lighting^d^. ADL score is derived using the formula CM = task duration + (number of errors × 2). Perceived difficulty is also evaluated.
Cellular Entry Search (CES)	Patient is required to find and read a specific entry on a 4-in. cellular phone within 10 s ^b^ in an environment with ambient lighting^d^. CES score = Task duration. Perceived difficulty is evaluated.
Reading Computer Screen (RCS)	Patient is required to correctly read text from a computer screen^c^ in an environment with ambient lighting^d^. ADL score is derived using the formula RCS = task duration + (number of errors × 2). Perceived difficulty is also evaluated.
Drops Bottle reading (DR)	Patient is required to correctly read the print on a typical bottle of eye drops within 20 s with task lightning^a^. ADL score is derived using the formula DR = task duration + (number of errors × 2). Perceived difficulty is also evaluated.
Subtitles reading (SuR)	Patient is required to correctly read movie subtitles on a computer screen^c^ with ambient lighting^d^. ADL score is derived using the formula SR = number of errors × 2. Perceived difficulty is also evaluated.
Open door test (ODT)	Patient is required to find a specific key from a keychain that holds 10 keys and open a door within 25 s with ambient lighting^d^. ODT score = Task duration. Perceived difficulty is evaluated.
Screwdriver test (ST)	Patient is required to select between three screws and three screwdrivers and insert each screw on a specific hole within 30 s with ambient lighting^d^. ST score = Task duration. Perceived difficulty is evaluated.

^a^Task lighting: 80 foot candles in workspace, 3000 K

^b^Cellular Screen Specifications: 640 × 1136 pixels (~326 ppi pixel density), 100% brightness

^c^Computer Screen Specifications: 21.5 in. screen, Times New Roman font size 12, 100% Zoom, 100% brightness, 50% contrast

^d^Ambient lighting: 70 foot candles, 3000 K

### Construction of activities of daily living. Validation study

Following construction of the ADLs, validation of the study framework was executed using a sample of 30 participants who visited our outpatient service in a consecutive-if-eligible recruitment method. These participants populated three validation groups according to their near vision acuity: a) Validation group J1 (VG-J1, 10 participants) with binocular near visual acuity Jaeger 1, b) Validation group J3 (VG-J3, 10 participants) with binocular near visual acuity Jaeger 3, and, c) Validation group J6 (VG-J6, 10 participants) with binocular near visual acuity Jaeger 6.

Test-retest reliability was assessed in the aforementioned validation groups by calculating intraclass correlation coefficients (ICCs) for all ADLs in two different visits with an average time-window of 1 month, to prevent memory effect. Construct validity was assessed with one-step analysis of variance (ANOVA) in order to confirm that all ADL tasks could efficiently discriminate validation groups based on their near visual acuity.

### Data collection

Within the ADL portfolio, PBS, SR, CES, and DR were a priori since these were especially demanding for near vision capacity and formed the subcategory of “difficult” ADLs (dADL). On the other hand, CM, CES, RCS and SuR reflect the minimal necessary near vision capacity of a citizen in a western society. Accordingly, they formed a separate subcategory (wADL) that was used for group comparisons. For all tasks, both ADL score and subjective difficulty were assessed. The latter was evaluated using 10 point-Likert scales (1 = maximal difficulty, 10 = no difficulty). In case a participant was unable to complete a task, the ADL score was not calculated; only the perceived difficulty was assessed. Overall ADL evaluation required an average of 30 min and was scored by an independent researcher blinded to the surgical technique performed to each participant.

Functional capacity of all participants was evaluated with the Visual Function Index-14 (VF-14) questionnaire, which was handed to the patients prior to their ADL assessment. In addition to the total score, two other VF-14 scores were calculated: a) Near vision VF score (VF-NV) derived from the items that assessed the perceived difficulty in near vision activities [items 1, 2, 3, 7, 8, 9 and 11, (reading small print, reading newspaper, reading a large-print book, doing fine handwork, writing checks, playing card games, cooking)], and, b) Distant vision VF score (VF-DV) derived from the items that assessed the perceived difficulty in distant vision activities [items 4, 5, 6, 10, 12, 13 and 14, recognizing people, seeing steps, reading traffic signs, taking part in sports, watching television, driving during day, driving during night)]. Among other clinical indexes, the following parameters were evaluated: a) binocular uncorrected distant visual acuity (BdUVA) using the Greek version of the Early Treatment Diabetic Retinopathy Study Chart at four meters distance then converted to a logarithm of the minimum angle of resolution (logMAR) acuity value to allow for statistical analysis, and, b) binocular uncorrected near visual acuity (BnUVA). All postoperative data collection was done within a 6 months timeframe starting from the patient’s last operation.

### Statistical analysis

The normality of measured data was evaluated by Kolmogorov-Smirnov test. Normal distribution data were assessed by Student’s *t*-test. Non-parametric data were assessed with the Mann-Whitney *U* test. Values at *p* < 0.05 were considered as statistically significant. All statistical analyses were performed with the Medcalc version 9.6.2.0 (Medcalc Software, Mariakerke, Belgium).

## Results

Results from the test-rest reliability analysis are presented in Table 2. All ADLs demonstrated ICCs above 0.90, suggesting high reliability. On the other hand, the ADL framework demonstrated sufficient construct validity since all ADLs could discriminate validation groups according to their near vision capacity (Table 3).

**Table 2:** Test-retest reliability assessment. Intraclass Correlation Coefficients

Parameter	Validation Study Participants		
	VG-J1	VG-J3	VG-J6
PBS (score)	97.92	94.97	94.18
PBS (dif)	97.51	98.13	97.88
SR (score)	97.68	94.94	98.65
SR (dif)	93.54	92.91	92.87
BR (score)	94.02	98.71	95.87
BR (dif)	96.44	96.33	93.12
CM (score)	94.41	93.25	92.48
CM (dif)	92.77	94.84	94.34
CES (score)	96.97	96.78	96.93
CES (dif)	97.91	96.63	95.04
RCS (score)	92.76	94.25	93.28
RCS (dif)	97.41	95.66	92.16
DR (score)	92.47	93.46	96.05
DR (dif)	90.77	91.83	91.11
SuR (score)	96.79	98.86	94.13
SuR (dif)	93.63	94.21	92.54
ODT (score)	96.86	91.52	90.26
ODT (dif)	92.36	93.15	91.43
ST (score)	91.51	92.31	93.39
ST (dif)	90.52	93.43	94.43

*PBS* = Phone Book Search; *SR* = Supermarket receipt; *BR* = Book reading; *CM* = Cellular message; *CES* = Cellular entry search; *RCS* = Reading computer screen; *DR* = Drops bottle reading; *SuR* = Subtitles reading; *ODT* = Open door test; *ST* = Screwdriver test; *score* = ADL score; *dif* = Perceived Difficulty

**Table 3:** Validity assessment of ADL framework

Parameter	Validation Study Participants					
	VG-J1		VG-J3		VG-J6	
		*SD*		*SD*		*SD*
PBS (score)^♭^	22.45	6.76	43.88	10.78	98.67	12.56
PBS (dif)^♭^	9.52	0.89	5.28	1.57	2.21	2.05
SR (score)^♭^	10.34	1.78	14.23	2.67	19.77	4.52
SR (dif)^♭^	9.06	0.47	6.34	1.28	2.41	1.87
BR (score)^♭^	41.41	12.46	67.76	14.23	121.89	23.65
BR (dif)^♭^	9.72	0.39	7.02	0.62	3.01	0.87
CM (score)^♭^	4.34	1.11	6.35	0.91	12.75	3.26
CM (dif)^♭^	9.88	0.24	7.24	1.13	2.11	1.84
CES (score)^♮^	5.87	0.69	8.76	1.42	14.74	2.62
CES (dif)^♮^	9.86	0.94	6.73	0.99	3.76	1.43
RCS (score)^♮^	12.21	1.85	15.26	2.53	21.62	3.61
RCS (dif)^♮^	9.76	0.46	7.89	0.71	4.34	2.65
DR (score)^♭^	9.14	2.98	18.74	4.66	37.63	5.65
DR (dif)^♭^	8.21	1.79	4.01	1.23	1.65	1.11
SuR (score)*	1.24	0.73	2.89	0.98	4.42	1.83
SuR (dif)*	9.79	0.21	8.32	0.73	6.99	0.52
ODT (score)*	12.33	1.63	15.12	1.02	18.88	1.43
ODT (dif)^♮^	9.74	0.58	8.27	0.38	5.35	0.99
ST (score)*	16.25	2.05	23.23	0.86	34.52	1.34
ST (dif)*	9.48	1.11	8.32	1.47	6.67	0.78

*PBS* = Phone Book Search; *SR* = Supermarket receipt; *BR* = Book reading; *CM* = Cellular message; *CES* = Cellular entry search; *RCS* = Reading computer screen; *DR* = Drops bottle reading; *SuR* = Subtitles reading; *ODT* = Open door test; *ST* = Screwdriver test; *score* = ADL score; *dif* = perceived difficulty

Values are one-way analysis of variance comparing scores among groups (*p < 0.05, ^♮^ *p* < 0.01, ^♭^ *p* < 0.001)

Regarding ADL performance, 122 patients [77 men and 45 women, 60.3 +/− 9.1 years] were recruited and populated MfG (60 participants) and MoG (62 participants) study groups. Non-significant differences could be detected in demographic data and the preoperative best spectacles corrected visual acuity (Table 4). As expected, significant postoperative improvement was detected in the BdUVA both for MfG [0.5 ± 0.09 (preop), 0.02 ± 0.06 (postop), *p* < 0.01] and MoG [0.52 ± 0.08 (preop), 0.04 ± 0.11 (postop), *p* < 0.01] participants. Although postoperative BdUVA differences were non-significant between groups (*p* = 0.09), MfG participants demonstrated significantly better BnUVA (*p* = 0.04) (Table 5). Better near vision in the MfG group was reflected as improved performance or less perceived difficulty in a series of ADL tasks (Table 6). Specifically, MfG participants performed significantly better in Phonebook Search (both task score and difficulty, *p* < 0.01), Supermarket Receipt (score: *p* = 0.04, difficulty: *p* = 0.02), and Drops Bottle Reading (score: *p* = 0.02, difficulty: *p* < 0.01) tasks. The Drops Bottle Reading task was identified as the most difficult by all participants, with 16.67% (10/60) of MfG and 85.49% (53/62) of MoG subjects being unable to complete it. In fact, MoG participants presented worse ADL scores and perceived more difficulty in almost all ADLs especially the demanding ones (dADLs). The latter resulted in: a) more MoG participants failing to complete those tasks (among them, 11.29% of MoG participants failed in Phonebook Search, 8.06% in SR, and, 9.68% in Cellular Entry Search), and b) MoG participants scoring significantly worse in the dADL subcategory (average dADL score: *p* = 0.02, average dADL difficulty: *p* = 0.01). On the other hand, non-significant differences could be detected in the wADL subcategory (both average wADL score and difficulty, *p* > 0.05). Moreover, overall ADLs score and difficulty was not significant among groups (average score: MfG: 15.59 ± 3.01, MoG: 17.93 ± 3.59, *p* = 0.07), difficulty: MfG: 8.67 ± 1.18, MoG: 7.99 ± 1.77, *p* = 0.08). Within this context, only VF-NV scores presented a borderline difference in favor of MfG participants (*p* = 0.05) while non-significant differences could be detected in the total scores for VF-14 and VF-DV (Table 5).

**Table 4:** Preoperative data for both study groups

Study group	No.	Age		BSCVA	
		years	SD		SD
MfG	60	61.7	8.9	0.50	0.09
MoG	62	60.1	9.2	0.52	0.08
*p*	NA	0.42		0.34	

*MfG* = Multifocal group; *MoG* = Monovision group; *BSCVA* = Best spectacles corrected visual acuity

**Table 5:** Postoperative group comparisons

Parameter	MfG		MoG		
	Mean	SD	Mean	SD	*p value*
Spheq (Dom)	0.21	0.17	−0.42	0.21	0.03
Spheq (nDom)	0.18	0.51	−1.35	0.31	<0.01
BdUVA	0.02	0.06	0.04	0.11	0.09
BnUVA	1.38	0.61	2.21	0.84	0.04
VF14 score	92.23	7.79	90.99	8.85	0.23
VF-NV score	93.15	3.92	89.48	5.62	0.05
VF-DV score	91.11	7.41	92.84	7.21	0.28

*MfG* = Multifocal group; *MoG* = Monovision group; *Spheq* = Spherical Equivalent; *Dom* = Dominant; *nDom* = Non-Dominant; *BdUVA* = Binocular Distant Uncorrected Visual Acuity; *BnUVA* = Binocular Near Uncorrected Visual Acuity; *VF 14* = Visual Function 14 questionnaire; *VF-NV* = Visual Function–Near Vision; *VF-DV* = Visual Function–Distant Vision

**Table 6:** Group comparisons for Activities of Daily Living

ADL		MfG			MoG			
		Mean	SD	NoP	Mean	SD	NoP	*p value*
PBS	*Score*	27.48	7.35	1	41.02	12.25	7	<0.01
	*Difficulty*	8.02	1.35		6.34	2.64		<0.01
SR	*Score*	11.29	1.31	-	13.98	2.04	5	0.04
	*Difficulty*	8.34	1.28		6.99	2.22		0.02
BR	*Score*	43.85	9.74	-	46.25	8.21	-	0.11
	*Difficulty*	9.04	0.88		8.75	1.75		0.09
CM	*Score*	5.25	0.84	-	5.32	1.34	-	0.19
	*Difficulty*	9.11	1.03		8.82	1.36		0.17
CES	*Score*	6.37	0.66	2	6.99	1.24	4	0.21
	*Difficulty*	9.12	0.65		9.01	0.77		0.19
RCS	*Score*	13.32	1.47	1	13.98	2.01	4	0.29
	*Difficulty*	8.46	1.99		8.78	2.08		0.34
DR	*Score*	12.96	3.02	10	17.46	2.53	53	0.02
	*Difficulty*	6.49	2.14		3.46	3.59		<0.01
SuR	*Score*	1.27	0.87	-	1.34	1.01	-	0.37
	*Difficulty*	9.32	1.19		9.17	1.45		0.24
ODT	*Score*	14.67	2.63	-	13.59	3.33	-	0.23
	*Difficulty*	9.56	0.44		9.48	0.75		0.41
ST	*Score*	19.45	2.18	-	20.41	1.89	-	0.18
	*Difficulty*	9.23	0.86		9.11	1.11		0.19
Average (all ADLs)	*Score*	15.59	3.01		17.93	3.59		0.07
	*Difficulty*	8.67	1.18	-	7.99	1.77	-	0.08
Average (dADLs)	*Score*	14.52	3.08		19.86	4.51		0.02
	*Difficulty*	8.01	1.23	-	6.44	1.89	-	0.01
Average (wADLs)	*Score*	6.55	0.96		6.90	1.41		0.09
	*Difficulty*	9.01	1.21	-	8.94	1.91	-	0.17

*MfG* = Multifocal group; *MoG* = Monovision group; *NoP* = Number of participants unable to perform task; *ADL* = Activity of daily living; *dADL* = Subgroup of ADLs (PBS, SR, CES, DR); *wADL* = Subgroup of ADLs (CM, CES, RCS, SuR); *PBS* = Phone Book Search; *SR* = Supermarket receipt; *BR* = Book reading; *CM* = Cellular message; *CES* = Cellular entry search; *RCS* = Reading computer screen; *DR* = Drops bottle reading; *SuR* = Subtitles reading; *ODT* = Open door test; *ST* = Screwdriver test

Correlation analysis was performed for ADL score, VF-NV score and near BnUVA. Both ADL score and VF-NV score demonstrated significant linear correlation with BnUVA (ADL r^2^ = −0.67, *p* < 0.01, VF-NV r^2^ = −0.55, *p* < 0.01). Furthermore, ADL and VF-NV scores presented excellent correlation (r^2^ = 0.53, *p* < 0.01).

## Discussion

Presbyopia is a common impairment in middle age adults with significant economic and psychological burdens [2, 16]. It affects billions of people at their productive age [17]. The majority of relevant studies identified presbyopia’s significant impact on the quality of life, especially in western societies [6, 16, 18]. The most prevalent correction method are the near-vision spectacles however, evidence suggests that even spectacles-corrected presbyopes suffer from significant reduction in their QoL, similar to systemic chronic diseases [7]. Therefore, it is no surprise that American presbyopes are willing to pay a premium of 5 USD per day to become spectacles-free for their near-vision disability [19].

Poor satisfaction rates for near-vision spectacles promoted intensive research for the surgical correction of presbyopia. Laser-assisted corrections [20–23], intrastromal rings [24], corneal inlays [25, 26] and lenticular approaches [27–29] attempt to address the presbyopia-related productivity loss and variable optical outcomes and satisfaction rates have been reported. Despite the variance of the published data, it is widely accepted that lenticular approaches offer an almost permanent surgical outcome for presbyopia since they address the ongoing or upcoming cataract changes of the lens as well, which are common in middle-aged and elderly presbyopes. On the other hand, corneal approaches (either laser-assisted or not) provide a short-term solution as these do not address the age-related changes of the lens.

In this study, we compared two prevalent lenticular approaches for presbyopia; pseudophakic monovision and bilateral multifocal lens implantation. Regarding our monovision patients, we selected the variable bilateral myopic defocus as described by our group recently [8] and compared their outcomes to the multifocal group that had bilateral implantation of the prevalent Restor diffractive intraocular lens with the +2.50 D add. However, apart from the conventional means of evaluating our surgical outcomes (i.e., BnUVA, VF-14, etc), we aimed to evaluate the objective performance of our participants in a series of daily tasks that mandate near-vision capacity. That was considered as necessary since, in our daily clinical praxis, we detected a significant lack of agreement between observed performance and subjects’ reports; substantial bias in reporting was observed in the published trials. In fact, the importance of ADL assessment is confirmed by a series of theories such as the Activity Theory, which claims that ADLs are the best method to evaluate a person’s disability [30].

Within this context, a prerequisite of the study was to identify and construct those ADLs that better reflect near-vision performance following presbyopia correction. Unfortunately, an extensive literature review on a specific ADL methodology was not found. Therefore, an exploratory pre-study was initiated, which identified ten ADLs that could be used both for cross-sectional and prospective comparisons. Taking into account the cultural differences in former presbyopia reports [16], the tasks in this study addressed the needs of a citizen in a developed country. Apart from the commonly used ADLs like the book reading and the phonebook search, we constructed ADLs for use of cellular phone use and personal computer as well as reading television subtitles. These formed a distinct category of ADLs (wADL) that reflected common tasks that the average man/woman has to perform in a Western society. Special attention in the ADL construction was given to the task and ambient lighting conditions in the simulated environment; the latest recommendations of the Illuminating Engineering Society were adopted for the purpose of this study [31].

Following the construction of the ADL framework we proceeded to validate its reliability: a) provide consistent outcomes when near visual acuity does not change and b) sufficiently differentiate presbyopes according to their near vision capacity. Both reliability and validity tests suggested that our ADL study framework was valid for group comparisons. Moreover, we could evaluate the level of agreement between the objective performance derived from ADL scores and with subjective QoL scores derived from the VF-14 questionnaire.

Regarding our study outcomes, we detected non-significant differences in the average ADL scores between bilateral multifocal lens implantation and our mini-monovision technique. However, the monovision group demonstrated reduced performance in almost all ADLs, especially for the demanding ones. This average ADL performance resulted in a significant number of MoG participants that were unable to address a series of tasks, resulting in significant difference in the mean scores for the demanding ADLs vs. the MfG group. A different monovision strategy (i.e., full monovision) could perhaps provide better results. Overall, there was a 5-fold likelihood for a MoG participant to be unable to complete a demanding near vision task in comparison with a patient who received multifocal IOLs. On the other hand, both MoG and MfG participants performed equally well in the wADL category. Therefore, we can prospectively conclude that both mini-monovision and multifocal lens implantation provide adequate visual competency to the modern middle-aged citizen of a Western society for the majority of their tasks.

Our correlation analysis indicate that ADL scores demonstrated sufficient correlation with near vision acuity and VF-14 scores. However, since VF-14 was developed as a subjective instrument of functional impairment due to cataract, it cannot fully address the presbyopic patient. Therefore, VF-14 presented a borderline significant difference in favor of MfG participants with no further information with respect to how demanding the tasks were. However, we are convinced that VF-14 is a convenient questionnaire for subjective evaluation of surgical outcomes, at least in clinical settings.

Evaluation of potential visual disturbances or stereopsis was beyond the scope of this study. Both our group and a series of former investigators have already published their results [8, 27]. Moreover, official Greek guidelines for refractive lens exchange are yet to be introduced by the National Ophthalmological Society hence we only selected presbyopes with cataract. Therefore, our outcomes should be further validated in presbyopes with no cataract changes in their lenses.

## Conclusions

To our knowledge, this is first study that objectively compares multifocal lens implantation and mini-monovision in a simulated environment using a predetermined ADL methodology. Our study outcomes could provide the necessary methodological framework for group comparisons among participants with different socio-demographic profile, lifestyle or working mandates and reveal the most compatible surgical approach for their presbyopia. Studies with larger cohorts and/or a different set of ADLs are necessary to confirm our results and further evaluate the long-term efficacy of these prevalent surgical options.
